# A Case of Pseudo-Pseudo Meigs’ Syndrome Despite Optimized Immunosuppressive Therapy for Systemic Lupus Erythematosus

**DOI:** 10.7759/cureus.46753

**Published:** 2023-10-09

**Authors:** Varun Dang, Andrew Rofail, Jade M Bowman, Joshua Peloquin

**Affiliations:** 1 Internal Medicine, West Anaheim Medical Center, Anaheim, USA; 2 Medical Education, California University of Science and Medicine, Colton, USA

**Keywords:** ca-125, serositis, pleural effusion, systemic lupus erythematosus, ascites, tjalma syndrome, pseudo-pseudo meigs

## Abstract

Pseudo-pseudo Meigs' syndrome (PPMS), also known as Tjalma syndrome, is a rare complication of systemic lupus erythematosus (SLE), characterized by a triad of ascites, pleural effusion, and elevated CA-125 levels. We report a case involving a 74-year-old female with a prior history of SLE who presented with recurrent bilateral pleural effusions, elevated CA-125 levels, and mild ascites. Imaging showed no evidence of any mass or malignancy. In this case, the patient's presentation aligned with the diagnostic criteria for PPMS. Additionally, all other potential causes were investigated, and no alternative pathologies better explained the patient's presentation. PPMS should be considered in the differential diagnosis when evaluating patients with this triad of symptoms and laboratory and imaging findings. Early and more accurate diagnosis can guide research into treatment modalities.

## Introduction

Pseudo-pseudo Meigs' syndrome (PPMS) is a rare condition characterized by a triad of ascites, pleural effusion, and elevated CA-125 in a patient with systemic lupus erythematosus (SLE) [1,2]. While this presentation may raise suspicions of malignancy, PPMS is not associated with either benign or malignant tumors. It is a diagnosis of exclusion once thorough testing has been done to eliminate other significant causes of these findings, including ovarian carcinoma or other cancers, liver cirrhosis, tuberculosis, congestive heart failure, and nephrotic syndrome, among others [3]. Typically, PPMS symptoms have a gradual onset, and the condition is most often diagnosed in patients with no known prior history of SLE [2]. The prevalence of this condition is currently unknown. Good prognostic outcomes have been seen with corticosteroids, immunosuppressants, or a combination of both.

## Case presentation

**Figure 1:**
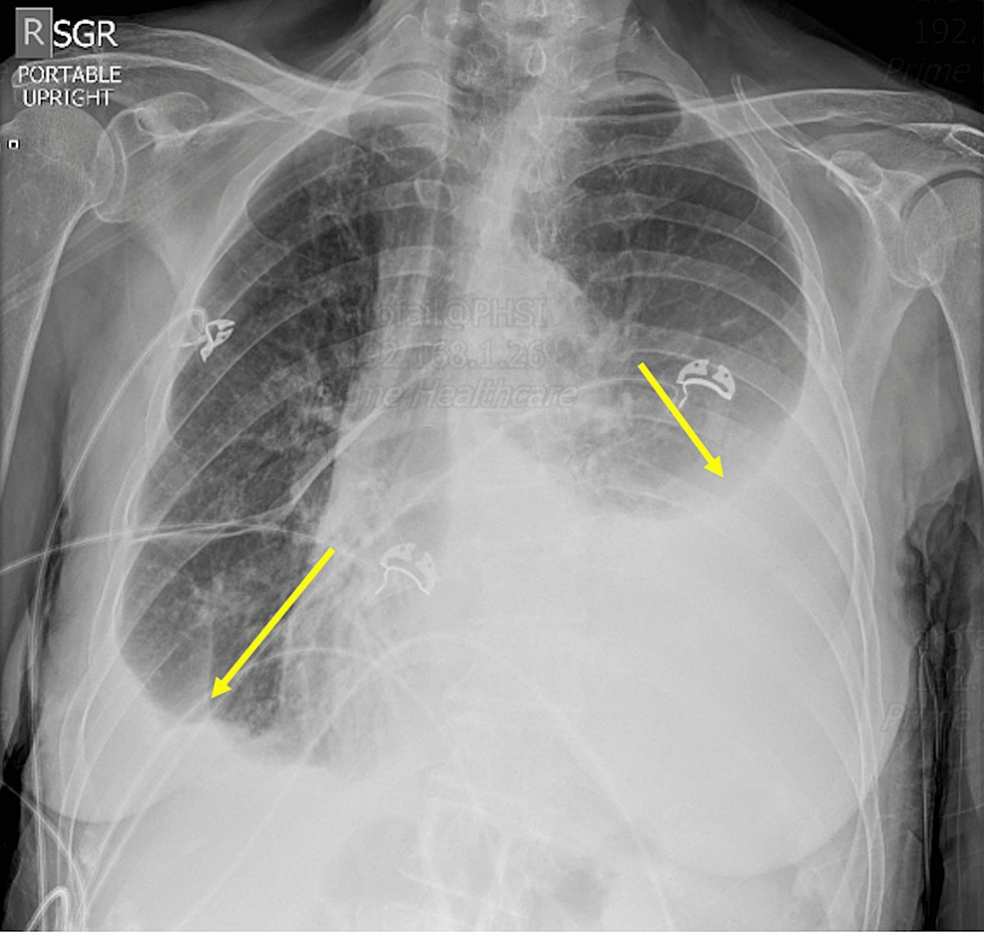
Chest X-ray showing bilateral pleural effusions.

A 74-year-old female with a past medical history of bronchiectasis, Helicobacter pylori gastritis, gastroesophageal reflux disease (GERD), hypertension, and known SLE confirmed by positive anti-nuclear antibody (ANA) and double-stranded DNA (dsDNA) testing in 2016, presented with a chief medical complaint of a three-day history of worsening abdominal pain, nausea, and shortness of breath. Relevant clinical presentations were present at the time of diagnosis, including myositis, inflammatory polyarthritis, anemia, and pleurisy with left-sided pleural effusion. The patient had tried multiple different medication regimens for SLE, but her most current regimen included mycophenolate (1.5 g twice daily), hydroxychloroquine (200 mg once daily), and methylprednisolone (4 mg twice daily). The patient's regular medications included pantoprazole for GERD/gastritis, clonidine and verapamil for hypertension, bronchodilators for bronchiectasis, and furosemide for lower extremity pitting edema. Physical examination was remarkable for decreased breath sounds in bilateral lung bases, tachycardia, 2+ pitting edema in bilateral lower extremities, mild tenderness to deep palpation in the epigastric area, and abdominal distension. The patient's oxygen saturation was around 86-88% on ambient air, and she was subsequently placed on two liters of oxygen via nasal cannula with an improvement of saturation to 96%. Initial laboratory workup and imaging were as follows: the renal function, coagulation profile, lipase, urinalysis, and liver function tests were all unremarkable. Complete blood count (CBC) showed normocytic anemia with hemoglobin of 9.7 (12-15.5 g/dL), but otherwise unremarkable. The erythrocyte sedimentation rate (ESR) was 50 mm/h (0-30 mm/h), and C-reactive protein (CRP) was 2.5 mg/dL (0.0-0.8 mg/dL). Her ANA was a 1:160 homogenous pattern (positive >1:80). Electrocardiogram showed sinus tachycardia. Chest X-ray showed bilateral effusions, moderate on the left and small on the right (Figure 1).

**Figure 2:**
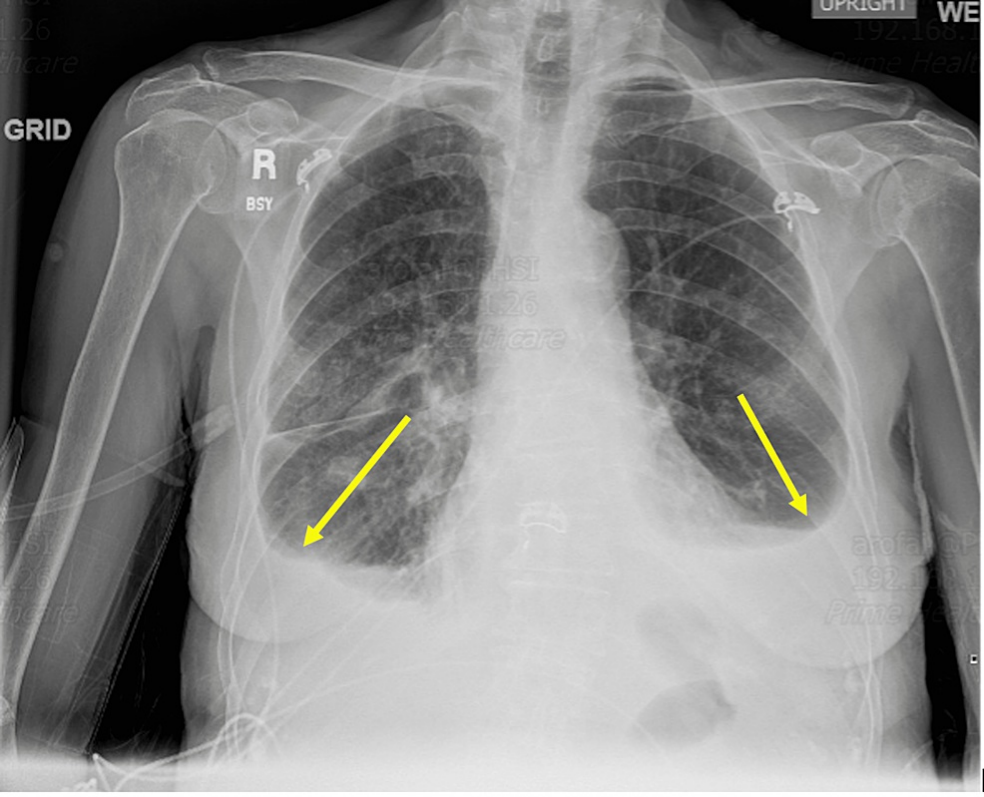
Chest X-ray showing reduction of left-sided pleural effusion after thoracentesis.

An abdominal ultrasound confirmed small ascites but was otherwise unremarkable, including the observation of a patent portal vein with no evidence of a clot. The echocardiogram revealed a hypertrophied left ventricle, an ejection fraction of 60-65%, mild diastolic dysfunction, trace mitral regurgitation, tricuspid regurgitation, and evidence of a large pleural effusion. A diagnostic and therapeutic left-sided thoracentesis was performed, during which 1.5 liters of yellow, cloudy fluid was removed. Subsequent to the thoracentesis, a chest X-ray was obtained, showing a marked reduction of the left-sided pleural effusion (Figure 2). Fluid analysis disclosed a total WBC count of 23, comprising 46% lymphocytes and 49% macrophages. At least one of Light's criteria (pleural fluid protein/serum protein >0.5) was met, with normal glucose and pH, indicating an exudative effusion consistent with Lupus pleural effusions. No aerobic or anaerobic organisms were cultured. Tests for adenosine deaminase were negative, and cytology and cultures showed no evidence of Mycobacterium tuberculosis. Furthermore, cytology revealed no indications of empyema, granuloma, or neoplasm.

**Figure 3:**
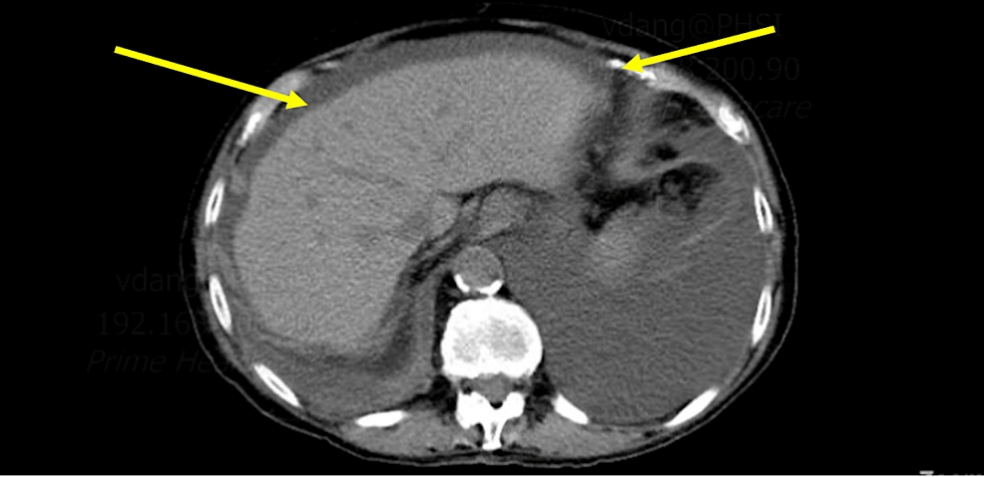
CT scan of the abdomen demonstrating ascites.

**Figure 4:**
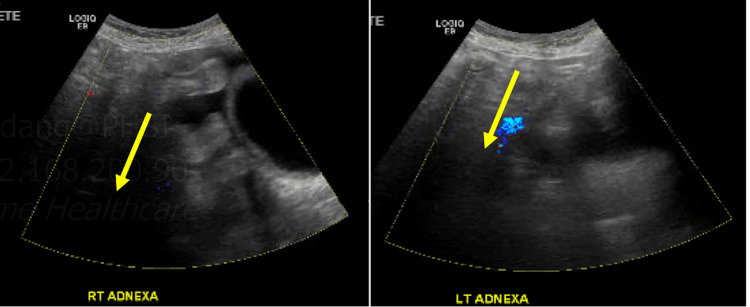
Pelvic ultrasound showing atrophic ovaries with no evidence of mass.

Further investigations were carried out to evaluate for underlying malignancy. The carbohydrate antigen 19-9 (CA 19-9) and carcinoembryonic antigen (CEA) were normal; however, cancer antigen 125 (CA-125) was elevated at 155 U/mL (0.0-38.1 U/mL). CT chest/abdomen/pelvis with IV contrast showed pleural effusions and ascites but did not demonstrate any signs of malignancy (Figure 3). Pelvic ultrasound revealed an atrophic uterus, ovaries, and small ascites but no evidence of ovarian malignancy (Figure 4). Esophagogastroduodenoscopy (EGD) demonstrated a small esophageal erosion, gastritis, and normal duodenum. Finally, the esophageal and stomach tissue biopsies revealed findings consistent with esophagitis/gastritis but no evidence of dysplasia or neoplasm. 

Based on our laboratory and imaging results, a diagnosis of PPMS was established. Following the drainage of the pleural effusion, the patient's respiratory status and symptoms improved significantly, and she was successfully weaned off oxygen supplementation. The patient was also administered per os (PO) antibiotics for pneumonia (PNA). Subsequently, she was discharged with a prescription for her home dose of hydroxychloroquine (200 mg daily), methylprednisolone (4 mg twice daily), and a reduced dose of mycophenolate (250 mg twice daily) to be taken during her recovery from PNA and throughout the completion of the antibiotic regimen. The patient was subsequently followed up by her rheumatologist two weeks after completing the antibiotic treatment. The dose of mycophenolate was resumed to 500 mg twice daily, and the patient demonstrated baseline oxygenation at that time, with no dyspnea even upon moderate exertion. Given her history of recurrent pleural effusions, it was recommended to repeat her SLE workup and obtain a consultation with a pulmonologist.

## Discussion

PPMS, also known as Tjalma syndrome, is a condition diagnosed by the triad of ascites, pleural effusion, and elevated CA-125. The severity of serositis and subsequent ascites seen in these patients has been postulated to be related to the degree of CA-125 elevation [4]. CA-125 is thought to activate mesothelial cells in patients with SLE. CA-125 increases the expression of growth factors such as VEG-F, especially in conditions such as pregnancy, ascites, menstruation, nephrotic syndrome, endometriosis, and leiomyomas [4-5]. It is also now being used as a promising biomarker in congestive heart failure prognostication. In our case, CA-125 was elevated with non-visualization of ovaries on ultrasound. The patient had no history of liver cirrhosis, congestive heart failure, chronic kidney disease, malnutrition, or ovarian pathology that could account for the presence of ascites. The presence of both pleural and ascitic fluid indicated polyserositis [6-7]. The diagnosis of PPMS requires fulfilling the following conditions: painless, gradual onset of polyserositis; elevated CA-125 in a patient with SLE; exclusion of benign or malignant tumors; and exclusion of polyserositis secondary to SLE complications, such as lupus nephritis and lupus peritonitis [6]. After excluding other diagnoses and noting a positive 1:160 ANA, homogeneous pattern, the patient met the SLE classification per EULAR/ACR criteria. Moreover, our patient met all three aforementioned criteria for the diagnosis of Tjalma syndrome [3].

As stated above, our patient had an exudative pleural effusion. Cytology of the pleural fluid showed mild lymphocytosis with no evidence of neoplasm, empyema, or granuloma. Fluid analyses for tuberculosis were also negative and, therefore, not suggestive. Alpha-fetoprotein (AFP), CEA, and CA 19-9 tumor markers were all negative. Given the absence of tumors in our patient, as seen in Meig's syndrome, our case may represent a previously unrecognized presentation of PPMS. In documented cases of PPMS, it was shown to be the initial presentation of SLE [1,8-9]. Our patient had several episodes of pleural effusions after her diagnosis of SLE in 2016, which could represent a previously unrecognized presentation of PPMS as a unifying diagnosis. PPMS has no association with any tumor; therefore, treatment is often difficult as removal of the offending agent is impossible. Pericardial effusion, pleural effusion, and ascites have all been previously documented [10]. All cases of remission in those with pleural effusion with ascites were achieved through treatment with immunosuppression with DNA synthesis inhibitors and steroids [11]. Our patient continued on previously prescribed immunosuppressive therapy, as stated, during the length of her hospital stay with resolution of symptoms after thoracentesis. Patients with active SLE and elevated CA-125 are often more likely to have serositis and pulmonary involvement. The pathophysiology is unclear, but it is thought that PPMS is due to immune complex deposition, aggregation of plasma cells, and vasculitis of mesenteric vessels leading to the various presentations of effusions seen in the literature [5,10]. 

## Conclusions

It is known that tumor markers may not always be associated with malignancy. Instead, in the setting of SLE, inflammation can cause serositis, which is thought to be the etiology of the unique clinical manifestations of PPMS. It also shows that PPMS can occur despite optimal immunosuppressive therapy for SLE. Our case highlights the necessity of increased research into treatment modalities in those with SLE with PPMS, as increased hospitalizations can incur a poorer outcome in these patients. More dedicated workup in those with SLE, especially those with polyserositis, can help diagnose PPMS earlier and help provide further insight into more targeted regimens needed for treatment. 
